# A Survey of Older Adults’ Self-Managing Cancer

**DOI:** 10.3390/curroncol29110634

**Published:** 2022-10-25

**Authors:** Kristen R. Haase, Schroder Sattar, Sandeep Dhillon, Heather M. Kilgour, Jennifer Pesut, Doris Howell, John L. Oliffe

**Affiliations:** 1 School of Nursing, University of British Columbia, Vancouver, BC V6T 1Z4, Canada; 2 College of Nursing, University of Saskatchewan, Saskatoon, SK S4T 0H8, Canada; 3 Department of Gerontology, Simon Fraser University, Vancouver, BC V6B 5K3, Canada; 4 BC Cancer, Vancouver, BC V5Z 4E6, Canada; 5 Department of Supportive Care, Princess Margaret Cancer Research Institute, Toronto, ON M5G 0A3, Canada; 6 Department of Nursing, University of Melbourne, Melbourne 3010, Australia

**Keywords:** cancer, aging, self-management, survey design

## Abstract

Background: Older adults living with cancer can experience significant challenges in managing their cancer treatment[s], care, and health. Cancer self-management is much discussed in the research literature, but less is known about the perceptions and experiences of older adults’, including their self-management capacities and challenges. This study explored the factors that supported and hindered cancer self-management for older Canadian adults living with cancer. Methods: We conducted a 17-item population-based telephone survey in the Canadian province of British Columbia among older adults (age ≥ 65) living with cancer. Descriptive and inferential statistics were used to analyze quantitative data and thematic analysis for open-text responses. Results: 129 older adults participated in the study (median age 76, range: 65–93), of which 51% were living with at least one other chronic illness. 20% reported challenges managing their cancer treatment and appointments, while only ~4% reported financial barriers to managing cancer. We organized the findings around enabling and encumbering factors to older adults cancer self-management. The main encumbering factors to self-management included health system and personal factors (physical and emotional challenges + travel). Whereas enablers included: access to interpersonal support, helpful care team, interpersonal support and individual mindset. Conclusions: Considering factors which enable or encumber older adults’ cancer self-management is critical to supporting the growing aging population in the work required to manage cancer treatment and navigate cancer services. Our findings may guide the development of tailored resources for bolstering effectual self-management for older Canadians living with cancer.

## 1. Introduction

Older adults aged 65 and older make up nearly half of all new cancer cases worldwide [1,2]. As people in developed countries continue to live longer, the number of older adults (>65 years-old) will also increase. With an aging population coupled with advanced cancer screening and early detection, it is estimated that by 2035 the incidence of new cancer cases among older adults will double, accounting for 58% of all new cancer diagnoses [1]. Older adults are distinct in many ways, including that they often have comorbidities and require tailored supports to manage their cancer treatment and care [3,4,5]. Older adults’ chronic illnesses may include diabetes, heart disease, dementia and orthopedic/functional challenges-which have been shown to increase toxicities from cancer treatments, negatively impact treatment, quality of life, and recovery [5,6]. Additionally, older adults take an average of 6.7 medications and require extended time with health care providers to discuss polypharmacy and other medication side effects [5,7].

However, older adults living with cancer value autonomy and the ability to stay functionally independent as individuals, and interestingly some of those with multiple comorbidities feel better equipped to deal with their cancer than their preexisting ailments [7]. Despite motivation for independence and autonomy in their lives and care, older adults can face challenges as they manage their cancer and treatment.

With an increasing demand on health care systems, people with cancer are encouraged to take an active role in their health by engaging in self-management [8,9]. Cancer self-management is defined as the collaborative partnership that occurs between health care providers and people living with cancer to facilitate a person’s ability to manage the acute and chronic effects of cancer and/or cancer treatment[s] [10,11]. Simply put, cancer self-management is what a person does to manage their own health with the support of health care providers and caregivers. This is especially important for the older adult population, where complex health needs due to comorbidities [12], polypharmacy [13] and additional supports [14] may be missed or mismanaged due to time constraints and an overburdened health care system [6,15].

Self-management is well-known in chronic disease management such as diabetes and hypertension, with tailored programs demonstrating improvements in mental health and other clinical outcomes [16,17,18]. However, older adults engaging in self-management can face challenges related to personal and system-level factors and structures. Health literacy is known to impact individuals’ capacity to self-manage chronic conditions; however those with the lowest health literacy are among individuals over 65 [15]. Existing research also shows that adequate time for discussion with health care providers is an important yet often missed aspect of teaching self-management in this population, as their multiple needs are often more complex [19]. In an effort to build on ongoing research and advance existing knowledge, the purpose of this article is to explore further the experiences of self-management—with a focus on factors that make self-management easier or more difficult- among older adults living with cancer.

## 2. Materials and Methods

We carried out this cross-sectional survey with support from the Canadian Hub for Applied and Social Research (CHASR) at the University of Saskatchewan. The survey was based on our prior work in the area, and guided by the expertise of our study team. The study population included community-based older adults with cancer living in the province of British Columbia.

### 2.1. Recruitment Procedure and Consent

Residents of British Columbia aged 65 and above were contacted using a random-digit dialing (RDD) method [20] that randomly contacted both land lines and mobile phones within the province. Contacted households were asked to identify if an older adult aged 65+ lived in the household, and if so, if they had been diagnosed with or treated for cancer in the last 2 years. Those households where no one answered the phone, or a person over 65 without cancer answered, were deemed ineligible. Individuals meeting the study criteria were read a disclosure about the study and asked to provide their informed verbal consent.

### 2.2. Data Collection

A 17-item survey including open and closed ended questions collected respondent demographic characteristics (age, gender, health status); cancer treatment information (cancer diagnosis; cancer treatment types; cancer stage); challenges and supports related to managing cancer; facilitators to managing cancer diagnosis; difficulties of cancer management; and financial barriers to cancer management. 

### 2.3. Data Analysis

Quantitative data were analyzed using IBM SPSS Statistics for Windows, Version 27 (IBM Corp., Armonk, NY, USA). Descriptive statistics (e.g., median, IQR, frequencies, percentages) were used to describe patient characteristics where appropriate (e.g., age, gender, cancer site and stage, treatment type, having other illnesses, education) as well as having challenges managing cancer diagnosis. Univariate logistic regressions were used to explore potential associations between age, gender, and having challenges managing cancer (yes/no). Answers to free-text questions were grouped based on thematic categories by two authors (HK, SD) and summarized in the method described by Braun and Clarke [21]. We have also quantified the occurrence of themes and sub-themes within the descriptive analysis. Although this is a departure from Braun and Clarke’s method, we feel it adds additional context. Developing themes were discussed with the first author, and refined through an iterative process in consultation with the team.

## 3. Results

An interviewer made contact with a total of 436 households who may have been eligible for the study, of which 129 participated. Of those who refused 200 did not provide a reason, and 107 reported a language barrier (response rate 29.5%).

Of the 129 older adults who participated in the study, the median age was 76 (IQR 11), range: 65–93. The majority of participants were white (91.5%) and the majority (66%) had some College/University-level classes or had completed a college or university degree (See Table 1 and Table 2).

The most common cancer types reported were prostate (24%), breast (14%), melanoma (11.6%) and colorectal cancer (8.5%). Nearly 60% of participants had received cancer treatment within the last 12 months and the most common treatment types were chemotherapy (14.7%), radiation (13.2%), and surgery (24.8%). The majority of participants (66.7%) did not know the stage of their cancer.

Half (51%) of participants were living with at least one other chronic illness. Only ~4% reported financial barriers to managing their cancer. Although only 20% reported having challenges in managing their cancer and treatment, 70% of the participants went on to describe one or more challenges or encumberances to cancer self-management. Amongst our sample, exploratory analyses indicated age (OR 1.06, 95 CI: 0.99–1.13) and gender (0.91, 95% CI 0.38–2.15) were not associated with having challenges in the self-management of their cancer.

### 3.1. Thematic Analysis

**Figure 1:**
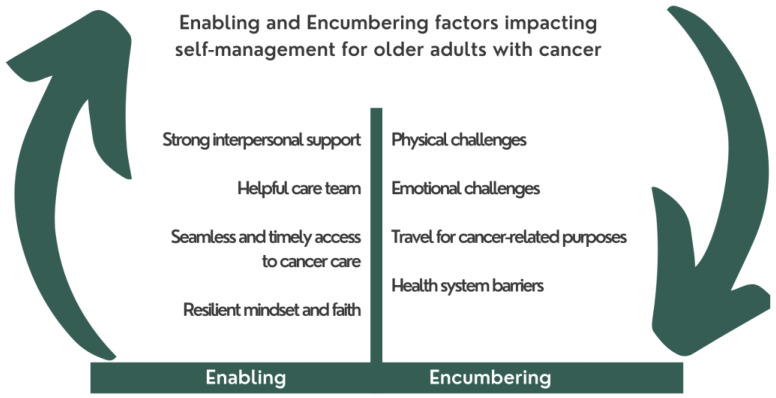
Enabling and encumbering factors impacting self-management for older adults with cancer.

Through our descriptive thematic analysis we analyzed open text responses to the questions: (1) What has made managing your cancer easier?; (2) what has made managing your cancer more difficult? And (3) Can you describe any financial limitations you have experienced? We framed these around two main themes related to factors that enabled or encumered self-management. The main encumberances to self-management included physical and emotional challenges, travel, and health system barriers. While enablers included external support from both healthcare and interpersonal social supports; seamless system processes; and resilient mindset and faith (Figure 1). These themes are reviewed below.

#### Encumbrances

**Figure 2:**
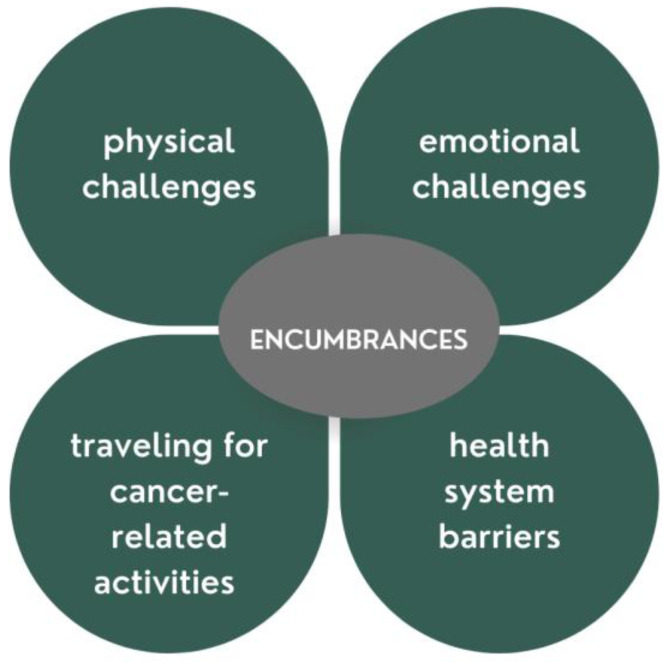
Encumbrances to self-management for older adults with cancer.

Participants were asked: what has made managing your cancer more difficult? Of the 129 participants, 32 (25%) reported that nothing had made managing their cancer more difficult. However, a total, 92 (71%) of participants specified at least one encumbrance that made their cancer self-management difficult. The four main themes that described fundamental encumbrances relate to the following: (1) physical challenges; (2) emotional challenges; (3) travel for cancer-related activities; and (4) healthcare system barriers (Figure 2).

(1) Physical challenges to cancer self-management

Twenty-four (19%) participants reported that physical challenges associated with their cancer and cancer treatment[s] made managing their cancer more difficult. Participants reported experiencing physical challenges associated with side effects from their treatment[s]. One participant shared: “*The side effects of the chemo stuff, it’s been pretty rough*” (67-year-old man with liver cancer). Another participant stated: “*At first, it was just going for the treatment and now managing nausea from the treatment*” (80-year-old woman with colon cancer).

Several participants reported fatigue as a particularly challenging side effect that impacted their cancer self-management. Fatigue often made activities of daily living challenging, resulting in some participants grieving the loss of activities they had enjoyed pre-cancer diagnosis. One participant mentioned: “*I think it’s the tiredness… I’m not moving as much. I’m not going out as much. I miss moving and going for walks. I used to garden a lot, do painting outside, but I don’t do much of that at all*” (80-year-old woman concurrently managing cancer, arthritis, diabetes, and mobility issues). Pain was the second most commonly reported physical challenge related to cancer self-management. This pain could be all consuming, as one participant living with throat cancer explained: “*Pain… it affects everything, it affects eating, talking, everything*” (71-year-old man). Some participants described physical pain as being caused by the location of their cancer, whereas others mentioned that the pain was a side effect of their cancer related treatment[s] or other comorbidities.

(2) Emotional Challenges

Twenty-two (17%) participants reported that the emotional toll of cancer and its treatment[s] made managing their cancer more difficult. For some, the word ‘cancer’ carried a heavy emotional weight and resulted in participants not being able to focus on much else. Several participants described how uncertainties related to their cancer made managing their cancer more difficult. This uncertainty could be related to their cancer, test results, or fears related to cancer recurrence. One participant said that they felt emotionally challenged because: “*there is not any specific way for the specialist to tell you if the cancer is going to return or not*” (74-year-old man with prostate cancer).

The uncertainty resulted in many participants experiencing stress, anxiety, and fear. One participant described the uncertainty associated with waiting throughout cancer care as traumatic: “*Everything is just a waiting game. And that causes a lot of anxiety and stress in my opinion because you don’t know, they can’t tell you what kind of cancer you have, they can’t tell you until they have taken a biopsy of the surgery, so it’s all waiting. And then after the surgery, another two weeks of waiting to be able to tell you what cancer, did they find more cancer in your lymph nodes, all that kind of stuff. And that’s what’s so traumatic*” (67-year-old woman with breast cancer).

For some, the emotional toll of cancer was compounded by other life events, impacting their ability to manage their cancer treatment[s], diagnosis, and care. For instance, one participant described having to navigate their cancer treatment amid moving homes, making managing their cancer more difficult. One older adult reported feeling more socially isolated as he aged, resulting in difficulties managing his cancer. He shared: “*It’s just not very good, it’s tough. I live alone and I’m old, 89 now, last birthday. Life is difficult when you’re old and don’t have any support system*” (89-year-old man with prostate cancer).

(3) Travel for Cancer-Related Activities

Fifteen (12%) participants described that transportation or travelling a distance to receive treatment[s] across British Columbia made managing their cancer more difficult. Travel to appointments could be compounded by the frequency of the appointments, as one participant shared: “*[I] had to get 25 treatments in a different hospital, with an hour drive each way*” (73-year old man with colon cancer). Whereas other participants indicated that the long travel to/from their cancer appointments impacted their cancer-self management responsibilities. This included one participant who drove 4–5 h for each appointment.

Though many participants noted transportation or distance to receive treatment[s] was an encumbrance that made their cancer self-management challenging, several highlighted that it was not an insumountable inconvenience. One participant said: “*[I] see the doctors locally here and then go 288 km to Kamloops, it’s no trouble anyways, there was no real difficulties involved. I believe the treatment I got was good and punctual*” (91-year-old man with melanoma).

(4) Health System Barriers

Fifteen (12%) participants noted system-wide challenges related to accessing prompt healthcare for their cancer treatment[s], diagnosis, and care. Most older adults described considerable staffing shortages resulting in challenges in accessing cancer care. This led to some participants feeling like they had to advocate further for care, as one explained: “*Nobody right now, no doctor, no matter how good they are, seems to continue the thing on, you have to be the one to contact them all the time about whatever is the matter*” (92-year-old man managing skin cancer and chronic obstructive pulmonary disease [COPD]).

Some older adults felt that even when they had access to their healthcare team, there simply was not enough time to sufficiently address their concerns due to system pressures. One participant said: “*It is so difficult to see my doctor and I can only see my doctor for one thing at a time and for only 15 min. The GPs are not set up in a way that he can look at everything I would want him to*” (65-year-old woman with lymphoma).

The over-burdened healthcare system resulted in some participants experiencing delays. One participant shared: “*I was postponed 3 separate times in favor of more seriously ill patients*” (73-year-old man with adenocarcinoma). Summing up system-wide challenges, one participant simply stated: “*you can’t hurry the system, you are just in the system, and that is, I think, the scary part of it*” (67-year-old with breast cancer).

### 3.2. Enablers

**Figure 3:**
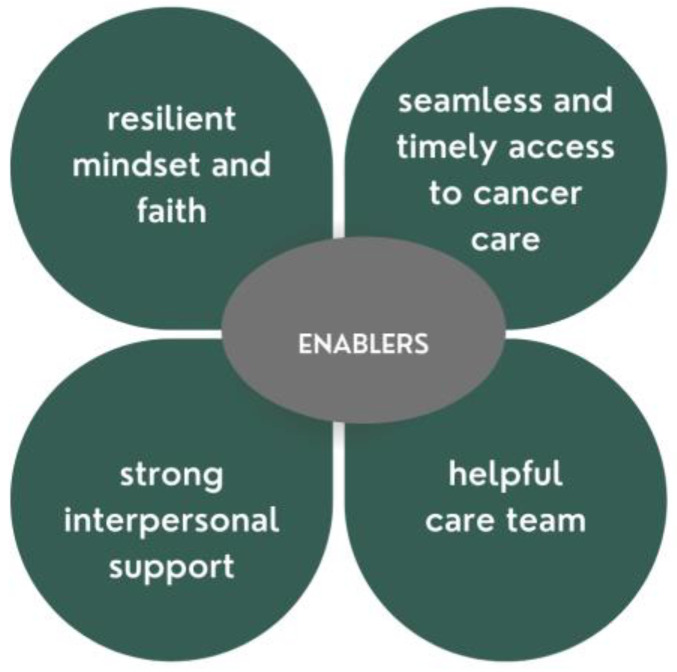
Enablers to self-management for older adults with cancer.

Participants were asked: what made managing your cancer easier? One hundred and twelve (87%) participants provided at least one enabling factor that supported their cancer self-management. The four main themes that described key facilitators relate to: (1) strong interpersonal support; (2) helpful care team; (3) effective treatment and screening; and (4) resilient mindset and faith (Figure 3).

(1) Strong interpersonal support

Nineteen (15%) participants stated that having strong interpersonal relationships, particularly with their family members and friends, were fundamental to managing their cancer treatment[s], care, and overall health. In particular, older adults highlighted having transportation to/from their appointments functioned as a primary facilitator. One participant stated: “*Probably all the help my wife gives me. She’s been a rock and takes care of all needs cause I’m not driving anymore either because [of the] pain meds that I take, and she does that*” (67-year-old man with liver cancer). Many participants mentioned the physical support of family and friends moving into their respective homes helped with their cancer self-management. One participant said: “*Having my sister-in-law moving in and looking after me and good friends and neighbors around me who were willing to come in and say,* “*Hi, how are you doing?*”” (81-year-old woman with breast cancer).

Other participants indicated the emotional support from their interpersonal relationships helped ease their cancer self-management responsibilities. One person mentioned: ”*My family has made it easier for me. They’re here for me all the time*” (76-year-old woman with breast cancer). Several participants stated that knowing they had supportive family and friends who could provide care helped them considerably. Whether it was the ability to speak to someone in their social network or transportation to cancer appointments, strong interpersonal relationships played an influential role in making their cancer experience more manageable.

(2) Supportive care team

Thirty-six (28%) participants felt that health care providers and cancer centres that were understanding and compassionate helped them to manage their cancer. Multiple participants stated that having access to their general practitioner and oncologist, along with a clear understanding of their treatment[s], enabled their self-management. One participant said: “*The availability of my GP, I can talk to him on the phone at any time*” (82-year-old woman managing skin cancer, heart disease, and breathing difficulties). Participants also emphasized how various cancer centres and clinics eased their experience navigating self-management responsibilities. This was consistently mentioned by older adults who lived in rural or isolated communities. One participant stated: “*the staff, and [the] access [I have] to things they offer, [including] online groups specific to my form of cancer. [I] live in [a rural area], so BC Cancer is working with the hospital––the wellness cancer section here, so I was able to stay in [my] home city for chemo because BC Cancer is working alongside the [local rural] hospital, making it closer for me*” (70-year-old woman with uterine cancer). Overall, several participants stated that a trusting and caring cancer team facilitated their cancer treatment[s], care, and health and facilitated their own abilities to manage their cancer diagnosis.

(3) Seamless and timely access to cancer care

Thirty-nine (30%) participants described receiving prompt treatment[s], referrals, and early detection acted as facilitators in supporting their cancer self-management. Participants repeatedly voiced the timely responses from their health care providers in accessing immediate treatment and referrals for surgery. One participant stated: “*It was [a] very quick response, and I was referred immediately to a surgeon and follow up with a good oncologist at the end of September. From there, it’s been quarterly blood tests and annual colonoscopy and scans*” (68-year-old woman with colon cancer). In these scenarios respondents’ self-management was buoyed and somewhat eased by the responsiveness of professional care and follow-up

Access to proactive practitioners that took time to listen to older adults about their health were additional facilitators participants mentioned in easing their self-management responsibilities*:* “*My urologist has been very proactive. He’s been very proactive about getting me to have the prostate removed and then initiating the radiation treatment*” (68-year-old man with prostate cancer).

Similarly, participants mentioned that scheduling screening tests regularly and promptly initiating treatment[s] made their cancer experience become more manageable. One participant stated: ”*I think early diagnosis in the sense that my family doctor noticed it and agreed that it should be looked at by a specialist*” (72-year-old man with basal cell carcinoma). Several participants noted that having surgery and knowing that the cancer treatment[s] ultimately helped reduce the need for their self-management. For instance, a participant said: “*You know, once you know that you are okay, that the cancer is gone, you feel more relieved, and eventually you just take it as it comes*” (67-year-old woman with breast cancer).

(4) Resilient mindset and faith

Twenty-three (18%) participants believed that having a resilient mindset and strong faith contributed to their cancer self-management. Participants expressed the importance of maintaining good physical and mental health throughout their cancer experience. Strategies including exercise, having a healthy diet, and maintaining a positive attitude made many older adults feel like they had more control over their health. One participant stated: “*State of mind, I guess. I’m just living each day the best I can [by] keep[ing] a positive attitude, don’t let it become an issue in your life*” (80-year-old woman with lung cancer).

This mindset was further emphasized when some participants spoke about the meaningful role religiosity and spirituality provided them through their cancer treatment[s] and experiences. For instance, one participant expressed: “*I find that my faith in God is what helps me the most*” (72-year-old woman with leukemia). The act of ‘forgetting’ about cancer treatment[s], care, and health also served as a vital coping mechanism for several participants in their self-management responsibilities. Older adults mentioned that not having to think about their cancer made managing their cancer easier. More specifically, one participant stated, “*I just seem to forget about it, and that’s it*” (81-year-old man with a history of prostate and kidney cancer). In this way, some participants were self-managing their anxieties related to cancer by bracketing cancer as background in favor of living their life.

## 4. Discussion

In this survey, we report on survey findings from older adults with cancer describing their self-management encumbrances and enablers. The majority of respondents (70%) described encumbrances to managing their cancer which ranged from personal challenges that might not be modifiable (i.e., physical and emotional challenges) to cancer care system barriers that are potentially modifiable. Our findings point to a need to consider how we support older adults with cancer (and their caregivers) for the work required to manage cancer. We suggest that changes are required at the level of the cancer care provider and within broader primary care systems to support older adults as they navigate their cancer trajectory.

The enablers and encumbrances to self-management described by participants included individual, interpersonal, and system aspects. Many of these encumbrances to self-management could be overcome with support from clinicians to engage in core self-management skills of problem solving, decision making, behavioral self-tailoring, goal setting and action planning, partnering with health-care providers, and risk reduction/health maintenance [10]. Previous research and clinical guidelines indicate that individual aspects may be attended to by more proactive assessment of older adults’ physiological and functional wellbeing [22]; whereas system challenges require more structural consideration [23]. Preparing people with cancer to engage in the self-management work of cancer has been identified as a short-coming by both patients and clinicians, thus more work is required to understand how exactly to prepare clinicians to support patients [8,24]. For older adults with cancer, nuance and expertise is required given broad heterogeneity related to age-related function and physiological changes [25]. Our findings bear this out by indicating that physical and emotional challenges inhibit cancer self-management. Given the increasing number of older adults expected to be requiring cancer care services in the years to come [1], these findings help us to understand where to intervene to support and prepare older adults to engage in cancer self-management.

Another interesting finding is that while only 20% reported challenges managing their cancer diagnosis and treatment, 70% went on to describe encumbrances to cancer self-management. One can only speculate as to why older adults are hesitant to indicate challenges to self-management but it may be rooted in an effort to maintain independence, autonomy, and agency [26]. This finding emphasizes the importance of asking and exploring older adults self-management needs in ways that can bolster support without inferring a lack of independence or impeding their autonomy [27]. One approach to address this is offering proactive assessment of older adults functional and physical needs via geriatric assessment [22]. Such assessments are recommended as the gold standard of care for older adults over the age of 65 with cancer [28,29] though widespread adoption has not been realized. Doing so has been shown to identify vulnerabilities related to some of the cancer self-management barriers that disproportionally impact older adults including travel, caregiver support, polypharmacy and care coordination. Such assessments will also provide insight into self-management capability and allow healthcare providers to understand when additional support for self-management is required. Addressing potential vulnerabilities often leads to changes in treatment plans [30], which may make the cancer journey more manageable for older adults.

Roughly half of the study participants reported having another chronic disease- which is lower than might be expected for a sample of this age [12]. However, the encumbrances shared by respondents draw out some of the concerns expected with the complexity of managing cancer as an older adult, for example, the emphasis on limited time with, and access to primary care providers. Participants detailed the mismatch between the restrictive one-issue per visit policy many primary care providers and the complexities of their health situation. These findings point to the need for collaborative and person-centered care- rather than siloed complaint focused care. Although BC (and much of Canada) are experiencing challenges related to primary care delivery, prior work has found that cancer for older adults delivered in the context of primary teams has promising impacts on chronic illness self-management [31,32]. These models can be emulated within the cancer care system- or better connection between cancer and primary care can be a future goal.

### Limitations

This study has several limitations, namely that the participants were predominantly white, English speaking and well-educated and not representative of the overall population due to our sampling approach (based on Statistics Canada data [33]). Our sample also reported fewer comorbidities than would be expected in a sample of older adults with cancer. We also did not know the proportion of those living in urban vs. more rural regions, which likely influences their access to care and other services. We used a RDD approach and were able to gather the perspectives of individuals across the province; however, we cannot safely exclude the possibility of response biases by virtue of our study design and recruitment strategy. Additionally, reporting response rates in RDD studies is problematic as the overall number of refusals/hang-ups/wrong numbers is difficult to track with certainty [20]. This study also has notable strengths, including the random sample of individuals from across the province, and the focus specifically on self-management in older adults which is an understudied area. Furthermore, the addition of open-ended questions provided illustrative insight into self-management experiences that survey questions alone would have left unaddressed.

## 5. Conclusions

Considering older adults’ cancer self-management strengths and capacities is critical due to the growing aging population. Understanding the multidimensional nature of self-management challenges, and key enablers are critical to ensure appropriate supports are made available. Notwithstanding the limitations of this study, our findings can begin to guide the development of tailored supports for cancer self-management in older adults.

## Figures and Tables

**Table 1:** Demographic Information.

Age (years)	Median 76 (IQR 11) Range 65–93 *
Women	67 (51.9%)
Race/ethnicity	
Caucasian	118 (91.5%)
Asian	2 (1.6%)
Latin American	1 (<1%)
Mixed racial background	1 (<1%)
Other	5 (3.9%)
Prefer not to disclose	2 (1.6%)
Highest level of education	
Some high school	8 (6.2%)
High school graduate	32 (24.8%)
Classes towards technical/college course/university or degree completion	86 (66.6%)
Total household income for 2021	
<$25,000	12 (9.3%)
$25,000–<$50,000	17 (13.2%)
$50,000–<$75,000	13 (10.1%)
$75,000–<$100,000	14 (10.9%)
$100,000–<$125,000	33 (25.7%)
Don’t know	16 (12.4%)
Prefer not to disclose	34 (26.4%)
Years since diagnosis	Median 2.5 (IQR 7) Range 0–45 **
Cancer sites	
Prostate	31 (24%)
Breast	18 (14%)
Skin	15(11.6%)
Colorectal	11 (8.5%)
Hematological	11 (8.5%)
Lung	8 (6.2%)
Gynecological	7 (5.4%)
Head and neck	6 (4.7%)
Bladder	5 (3.9%)
Kidney	4 (3.1)
Liver	3 (2.3%)
Pancreatic	1 (<1%)
Other	7 (5.4%)

* Out of 127 respondents reporting age. ** Out of 124 respondents reporting year since diagnosis.

**Table 2:** Sample characteristics.

On cancer treatment/received treatment in past 12 months	77 (59.7%)
Treatment type	
Chemotherapy	19 (14.7%)
Radiation	17 (13.2%)
Endocrine	13 (10.0%)
Surgery	32 (24.8%)
Other	26 (20.1%)
Have other illnesses	66 (51.2%)
Finances a barrier to cancer management	5 (3.9%)
Had challenges managing cancer treatment and appointments	26 (20.2%)

## Data Availability

The data that support the findings of this study may be available on request from the corresponding author. The data are not publicly available as they could compromise the privacy of research participants.
